# Single ulcers on the tongue dorsum: differential diagnosis between paracoccidioidomycosis and squamous cell carcinoma

**DOI:** 10.4317/medoral.26518

**Published:** 2024-04-14

**Authors:** Claudia Rebecca Costa Cavalcante, Maria Emília Mota, José Divaldo Prado, Oslei Paes de Almeida, Christianne Soares Moreira Barbosa, Joao Adolfo Costa Hanemann, Norberto Nobuo Sugaya, Maria Stella Moreira, Fábio Abreu Alves

**Affiliations:** 1 Department of Periodontics, ABO, Maceió, Alagoas, Brazil; 2 Department of Stomatology, School of Dentistry, University of São Paulo (USP), São Paulo, SP, Brazil; 3 Department of Stomatology, A.C. Camargo Cancer Center, São Paulo, Brazil; 4 Department of Oral Diagnosis, Piracicaba Dental School, University of Campinas (UNICAMP) Piracicaba, SP, Brazil; 5 Private clinic, Osasco, São Paulo, Brazil; 6 Department of Clinic and Surgery, School of Dentistry, Federal University of Alfenas, Alfenas, MG, Brazil

## Abstract

**Background:**

Paracoccidioidomycosis (PCM) is the leading cause of death among systemic mycoses in Brazil. On the other hand, oral squamous cell carcinoma (OSCC) is the most prevalent malignant neoplasm of the mouth. Both lesions rarely affect the tongue dorsum and may share similar clinical characteristics. This study aimed to retrieve cases of single oral ulcers diagnosed as PCM or OSCC.

**Material and Methods:**

A cross-sectional retrospective study was conducted. All patients who had a single ulcer on dorsum of the tongue and confirmed diagnosis of PCM or OSCC were evaluated.

**Results:**

A total of 9 patients (5 women and 4 men) were evaluated, 5 patients had OSCCs (mean age = 69,8 years old), and 4 patients PCM (mean age = 51 years old). Most of the lesions were infiltrated and indurated in the palpation exam. Duration ranged from 1 to 12 months (mean time of 5.2 months and 4.7 months for OSCC and PCM, respectively). OSCC was the main clinical diagnosis hypothesis.

**Conclusions:**

Although uncommon, PCM and OSCC should be considered as a diferential diagnosis hypothesis in infiltrated ulcers on the tongue dorsum. Iincisional biopsy is mandatory to confirm the diagnosis and indicate the appropriate treatment.

** Key words:**Paracoccidioidomycosis, oral squamous cell carcinoma, oral ulcer, tongue dorsum.

## Introduction

Paracoccidioidomycosis (PCM) is an endemic and neglected disease in Brazil, caused by *Paracoccidioides brasiliensis* and *Paracoccidioides lutzii* (1,2). It is accepTable that the main if not the only form of contamination occurs through the inhalation of spores (3-5). Most patients evolve as a primary pulmonary focus, developing a chronic infection. In some cases, hematogenous and/or lymphatic propagation occurs in other locations, such as skin, adrenal glands, central nervous system, bones, and oral mucosa (6).

Oral PCM manifestations consist of multiple moriform lesions/ulcers, with the gingiva and alveolar ridge mucosa responsible for 51% of the affected sites of the mouth (7). On the other hand, Oral Squamous Cell Carcinoma (OSCC), which is responsible for 90% of oral cancer, commonly presents as a single irregular ulcer in the lateral border of the tongue. It is well known that OSCC is related to intense and prolonged exposure to tobacco and alcohol, most in men over 50 years of age. However, in the last 3 decades, the epidemiological profile has been changing, with the diagnosis of patients younger than 45 years old and lesions originating from HPV infection (8).

Although lesions affecting the dorsum of the tongue are uncommon, some studies have described cases of amyloidosis, hamartomas, leiomyomatosis, syphilis, and OSCC (9-14). An interesting case of PCM affecting the dorsum of the tongue had OSCC as the main diagnosis hypothesis (14). Thus, the present study aimed to compare cases of single oral ulcers on the tongue dorsum diagnosed as PCM or OSCC and discuss its main features.

## Material and Methods

This cross-sectional retrospective study was approved by the Institutional Committee on Ethics (no 4.754.713). All cases presenting as a single ulcer on the dorsum of the tongue with a confirmed diagnosis of PCM or OSCC were evaluated. Clinical pictures (patients’ photography) were accessed to evaluate the clinical characteristics. Patients who had multiple PCM lesions or PCM/OSCC in other regions of the mouth were excluded.

Demographic data, habits, time, symptoms, diagnosis hypothesis, and stages were collected from patients’ charts.

## Results

A total of 9 patients, 5 women and 4 men, were evaluated. OSCCs were diagnosed in 5 (mean age = 69,8 years old, ranging from 58-88), and PCM in 4 patients (mean age = 51 years old, ranging from 41-72. Tobacco use was reported in all cases of OSCC, and alcohol in 2. Of the PCM patients, all 4 patients were smokers, 2 were alcoholics and 1 patient denied any habits (Table 1).

**Figure 1:**
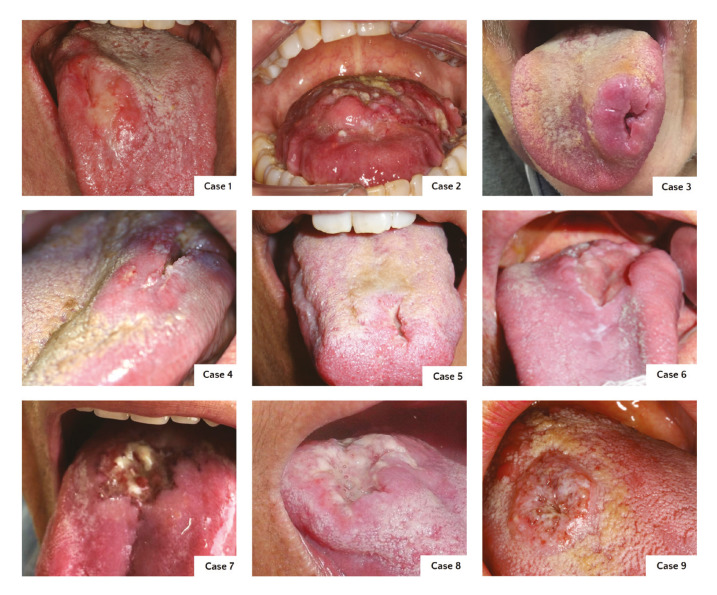
Oral squamous cell carcinoma (cases 1 to 5) and paracococcidioidomycosis (cases 6 to 9) on the dorsum.

Both OSCC and PCM patients presented a single ulcer on the dorsum of the tongue. Most of the lesions were infiltrated and indurated in the palpation exam, and duration ranged from 1 to 12 months (mean time of 5.2 months and 4.7 months for OSCC and PCM, respectively) (Fig. 1).

Pain was reported by 3 patients with OSCC and in only one with PCM. For all cases, OSCC was the main clinical diagnosis hypothesis and syphilis and PCM were also considered in the PCM cases. Two patients with OSCC were considered with advanced stages based on TNM staging (Table 1).

## Discussion

PCM is a clinical differential diagnosis of OSCC, mainly if PCM presents as a single lesion in the mouth (14,15), particularly on the dorsum of the tongue (14). Here, we are reporting additional 9 cases, 5 OSCC and 4 PCM, affecting the dorsum of the tongue. The similarity of the clinical features of both lesions is evident in the Figures here shown. All 4 cases of PCM had OSCC as the main diagnosis hypothesis, and PCM, tuberculosis, and syphilis were other options for clinical diagnosis. Moreover, some authors added histoplasmosis, leishmaniasis, syphilis, and Wegener's granulomatosis as other differential diagnoses of single oral lesions of PCM (16).

Oral mucosa is involved in 50% of the cases of PCM (2), and in general the lesions are multiple with moriform surface. Gingiva/alveolar ridge (23.2%), lips (21.7%), and buccal mucosa (15.9%) are the main locations. The tongue is involved in around 11.2% of the cases. The presence of single lesions, as in our cases, is extremely rare (7,14,16). Men are more frequently affected by PCM (19:1), between the ages of 30 and 60 years (3). Epidemiological studies indicate the rarity of PCM in women, especially during the reproductive years, which suggests a protective action of the female hormones (17). Although multiple lesions are the most common presentation of oral PCM as mentioned before, our findings shared the same features as those presented by de Oliveira Gondak, where a significant part of the patients who presented a single lesion were women (15). Therefore, further studies evaluating the association of single oral lesions with the influence of female hormones are needed.

OSCC on the dorsum tongue is extremely rare. In a Japanese study that evaluated 368 tongue SCC, only three cases affected the dorsum (0.8%) (18). In addition, such tumors had their diagnoses in advanced stage due to delay in suspecting of malignancy (18,19). Two out 5 cases of our series had advanced clinical stage. It is important to define the appropriate diagnosis of these single ulcers in the dorsum of the tongue to promptly establish adequate treatment. Both diseases, OSCC and PCM, may present similarities in the patient profile and lesion clinical features, however, the therapeutic modality is totally different. PCM is treated with prolonged antifungal medicines (1,3) and, in contrast, OSCC is treated with surgical approaches and other modalities are added in more advanced stages, such as radiotherapy and chemotherapy (20,21). Therefore, an early and correct diagnosis with histopathological analysis can prevent unnecessary invasive/radical treatment.

In summary, PCM and OSCC should be considered as diagnostic hypotheses in single and infiltrating ulcers on the dorsum of the tongue. After detailed anamnesis and clinical examination, an incisional biopsy is mandatory to define the diagnosis and appropriate treatment of single ulcers in the tongue dorsum. Finally, it is interesting to consider that our cases of PCM here described were more frequent in women, and this observation deserves to be better evaluated in future epidemiological studies.

## Figures and Tables

**Table 1:** Demographic data of 5 patients with OSSC and 4 with PCM presented as a single lesion on the dorsum of the tongue.

Case	Age	Sex	Tobacco/ alcohol	Time (months)	Symptoms	Diagnosis Hypothesis	Diagnosis	Stage
1	62	F	T/A	5	Pain	OSCC	OSCC	T2N0M0
2	65	F	T	12	Pain	OSCC	OSCC	T4N0M0
3	76	M	T	5	No	OSCC	OSCC	T2N0M0
4	58	M	T/A	1	No	OSCC	OSCC	T4N2bM0
5	88	M	T	3	Pain	OSCC	OSCC	T2N0M0
6	42	F	T/A	12	Pain	OSCC; PCM; Syphilis	PCM	NA
7	72	M	No	2	No	OSCC	PCM	NA
8	41	F	T/A	1	No	OSCC; PCM	PCM	NA
9	49	F	T	4	No	OSCC, Tuberculosis	PCM	NA

T = tobacco / A = Alcohol / / NA = not applicable
